# Investigation of antireflective and hydrophobic properties in polycrystalline GaN films with dual porosity produced by CVD

**DOI:** 10.1038/s41598-019-48202-4

**Published:** 2019-08-12

**Authors:** Josue Mena Gómez, Joan J. Carvajal, Oleksandr Bilousov, Francesc Díaz, Magdalena Aguiló

**Affiliations:** Universitat Rovira i Virgili, Departament Química Física i Inorgànica, Física i Cristal·lografía de Materials i Nanomaterials (FiCMA-FiCNA) - EMaS, Campus Sescelades, E-43007 Tarragona, Spain

**Keywords:** Spectrophotometry, Electronic devices, Electronic materials

## Abstract

We optimized the deposition conditions of polycrystalline nanoporousGaN coatings produced by Chemical Vapor Deposition on Si substrates, by exploring the effect produced by the Ga holder shape, the initial amount of Ga, the reaction deposition time and the metallic catalyst used. Such polycrystalline films probed to act as antireflective coatings by reducing the reflectance of Si substrates by 50% or more, and that of flat GaN samples by 40% in the UV and 83% in the visible, at the same time that they exhibit an almost constant reflectance from 400 to 800 nm, important to develop UV sensors with enhanced sensitivity. Furthermore, the polycrystalline nanoporous coatings we developed exhibit hydrophobic behaviour, with a static contact angle of 119°, and a contact angle hysteresis of 4.5°, which might contribute to enlarge the durability of such functional films, by the self cleaning effect induced.

## Introduction

Nowadays, antireflective coatings are common components used in windows, solar cells, display technology, sensors and eyeglasses to increase quantum efficiency and reduce light pollution^1,2^. Such coatings consist of optical films that reduce the reflection of light at optical interfaces, generated by a contrast of refractive indices. One of the most effective ways to avoid this effect is the generation of refractive index gradient structures^3^. However, they required to exhibit refractive indices around 1.22–1.23 when coating glass substrates^4^, a parameter not fulfilled by dense solids with refractive indices above 1.3^5^. In this context, porous materials with pore sizes in the subwavelength scale are an alternative to develop such antireflective coatings.

These porous materials might be also used to change the wettability properties of the surface of the material considered^6^. By controlling the porosity, a surface can change their properties from hydrophilic to hydrophobic^7^. Thus, combining the antireflective and hydrophobic properties it is possible to develop functional materials with improved properties for applications in self-cleaning antireflection coatings^8^, self-cleaning solar cells^9^ or sensors^10^.

The fabrication techniques used to produce such porous antireflective and hydrophobic coatings comprise methods such as spinning, spraying, dipping, electrochemical deposition, plasma etching or layer by layer deposition methods^5,11^. However, most of these methods suffer from complicated procedures, long deposition and aging times, in compatibility with organic components, repeated processing procedures and of expensive devices to produce these antireflective coatings, to name a few^5,11^. Furthermore, the nanopores generated can suffer from being filled by water molecules present in the ambient gas via capillary condensation^12^, or from a limited diffusion of the functional material into the pores^13^, which in any case, reduces the antireflective effect. Nanopores also might be blocked when using postsynthetic grafting procedures, resulting in a reduction of the porosity degree and consequently in an increase of the refractive index^5^. Another inconvenient to be taken into account is the need, in some cases, of high temperature calcinations to remove organic materials or templates, with the potential loss of functionality^5,11^. Thus, new low-cost preparation techniques and materials are required to produce mechanically and chemically robust, high-performance, and if possible, multifunctional antireflective coatings.

Wurtzite gallium nitride (GaN) is a direct wide band gap semiconductor with important applications in opto-electronics, such as light emitting and laser diodes, high-power and high-frequency devices, and recently, chemical and biosensors^14^. Antireflective and hydrophic surfaces in GaN have been demonstrated in GaNnano-flowers fabricated by Chemical Vapor Deposition (CVD)^15^, and in N-polar GaN surfaces nanostructured by wet photochemical etching under UV illumination and chemically functionalized with lauric acid^16^. Furthermore, the wetting properties of GaNmicrobelts with rough surfaces synthesized by CVD^17^, and etched Ga-polar GaN surfaces functionalized with 1-dodecanethiol^18^ have also been investigated. Recently, by attaching a polymer layer with an array of micropyramidal structures on GaN, antireflective and hydrophobic structures have also been demonstrated for sensing purposes^10^. So, it is clear that it exists an increasing interest in developing antireflective and hydrophobic structures in this material.

We demonstrated the possibility of producing nanoporousGaN by using the direct reaction between metallic Ga and NH_3_ in a simple Chemical Vapor Deposition (CVD) system^19^. With our approach, we produced nanoporous GaN in one single step, without requiring any post-growth treatment to induce the porosity^20^. Furthermore, this technique allowed for an easy integration of nanoporousGaN on Si substrates^21^.

In the present work, we optimized the deposition conditions for nanoporous GaN, obtained by CVD, to produce antireflective polycrystalline films on Si substrates. These antireflective coatings reduced the reflectance of Si substrates, metal-coated (Au and Pt) Si substrates and GaN by 40% or more, depending on the wavelength region analyzed, improving the performance of the currently developed antireflective coatings technology especially for short wavelengths, below 400–500 nm. Furthermore, these polycrystalline nanoporous films probed also to be hydrophobic, contributing to generate multifunctional layers with improved properties for applications in different fields, but especially as UV sensors.

## Experimental Section

### Deposition of polycrystalline nanoporous GaN

**Figure 1:**
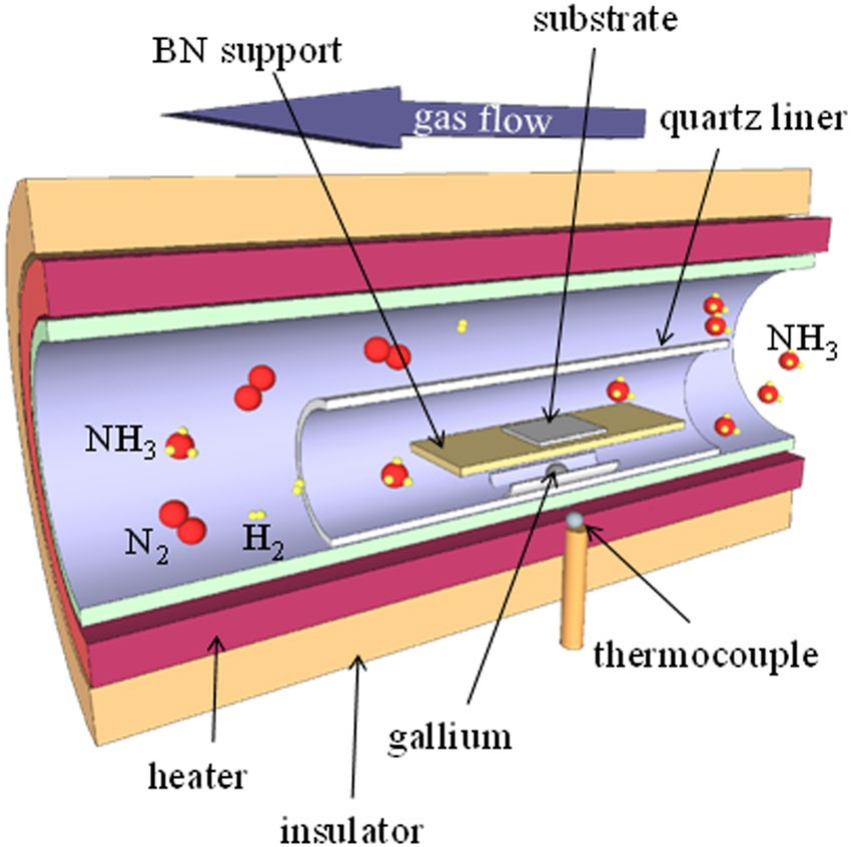
Schematic representation of the CVD system used for the deposition of nanoporous GaN films on Si substrates.

Nanoporous GaN particles were grown on metal-coated Si substrates by the direct reaction of metallic Ga (Alfa Aesar 99.999%) and NH_3_ (CarburosMetálicos >99.98%) as Ga and N sources, respectively, in a CVD horizontal tubular furnace. A scheme of the CVD set up is shown in Fig. 1.

Metallic Ga was introduced into the system in the form of a drop placed on a quartz crucible. The quartz crucibles or Ga holders used had three different shapes to limit the spreading of liquid Ga along different directions: (i) a flat plate, where the spreading of liquid Ga is not restricted; (ii) the half of a cylindrical tube, where the spreading of liquid Ga is allowed only along the axis of the tube; and (iii) a concave crucible, where the spreading of liquid Ga is restricted in all directions.

As substrates, we used silicon wafers with (100) and (111) crystallographic orientations. The Si substrate was placed above the Ga source at a vertical distance of 2 cm, supported by a BN plate. Prior to their introduction in the furnace, the silicon substrates were cleaned with piranha solution and ethanol.

We tested the effect of different metallic catalysts on the formation and the structure and morphology of the polycrystalline coatings on the Si substrates. We used Ni(NO_3_)_2_, deposited on the surface of the substrate by spreading several drops of an ethanolic solution of the compound used and continuous films, 20 nm thick, of Ni, Au, Pt, and Ti, deposited on the substrates by RF sputtering (AJA International) at a power of 150 W and a pressure of 3 mTorr. We selected this thickness for the metallic films owing to the impossibility of forming thinner continuous and uniform layers of Au on silicon. Afterwards, we kept this layer thickness constant in the rest of metals sputtered on silicon wafers for comparison.

In a typical experiment of deposition of polycrystalline nanoporous GaN films, the quartz reactor, 5 cm in diameter and 1 m long, was degassed to a vacuum pressure of 1 × 10^−2^ Torr. Ammonia was then introduced through a mass-flow controller at 75 sccm from one end of the quartz reactor and the furnace was heated up to the reaction temperature of 1200 K. Then, the furnace was kept at this temperature during the reaction time, which we tuned between 30–120 min, under a constant flow of NH_3_; keeping the pressure of the system constant at 15 Torr while the chemical reaction was taking place. When the reaction was finished, we cooled down the furnace to room temperature and stopped the ammonia flow. These parameters had been optimized previously^19^.

### Nanoporous GaN coatings characterization

The aspect of the nanoporous GaN coatings deposited on the Si substrates was characterized using a JEOL JSM 6400 scanning electron microscope (SEM). Before observation, the samples were coated with Au in a Bal-Tec SCD004 sputterer.

X-ray diffraction (XRD) patterns of the samples were recorded in a Bruker AXS D8-Discover diffractometer equipped with parallel incident beam (Göbel mirror), a vertical θ-θ goniometer, and a General Area Diffraction Detection System (GADDS) HI-STAR with a multiwire proportional counter with an area of 30 × 30 cm and 1024 × 1024 pixel density. The X-ray diffractometer was operated at 40 kV and 40 mA to generate the Cu Kα radiation. Data were recorded from 20–90° in the 2θ range. The incident beam and the detector were placed at a distance of 15 cm of the sample. Identification of the crystalline phases obtained was achieved by comparing the XRD diffractogram with the Joint Committee on Powder Diffraction Standards (JCPDS) database using Diffrac^plus^ Evaluation software.

Focused ion beam (FIB) tomography was used to reveal the internal porous structure of the particles with a Zeiss 1540 Cross Beam microscope equipped with a FIB column with a Ga source and a high-resolution field emission electron column. The angle between the FIB and the electron columns was 52°, with the sample surface perpendicular to the ion column. 5 pA beam current was used for ion milling to minimize surface damaging and materials redeposition.

Reflectance measurements of the nanoporous GaN coatings were done in an Agilent Cary 5000 UV-Vis-NIR Spectrophotometer equipped with an Internal Diffuse Reflectance Accessory (DRA) working in a specular configuration, so both, specular and diffuse reflections were collected.

To evaluate the contact angle, a 2 μl water droplet was placed above the surfaces smoothly with a micropipette. The droplet profile was recorded using a video-based optical contact angle measuring goniometer OCA 15EC by NEURTEK Instruments.

## Results and Discusion

### Effect of the gallium holder’s shape

A set of experiments with these holders for Ga with different shapes were carried out to analyse how it affected the spreading of the Ga drop, and, consequently, which was the final shape and porosity of the coatings obtained.

**Figure 2:**
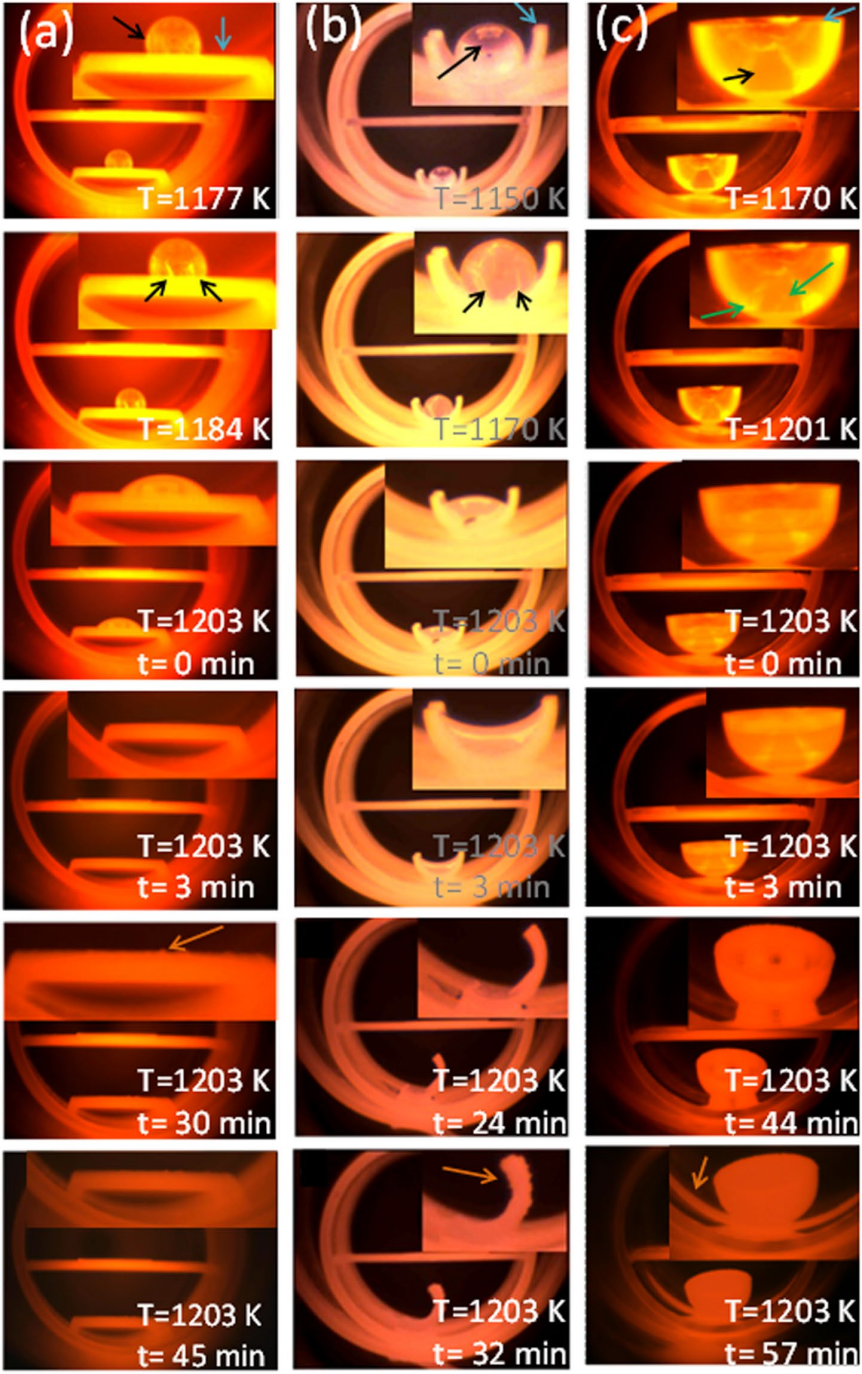
Optical images showing the evolution (cracks are pointed with a green arrow) of the Ga drop (pointed with a black arrow) on the different Ga holders (pointed with a blue arrow) used in these experiments: (**a**) flat plate, (**b**) half cylindrical tube, and (**c**) concave crucible.

Figure 2 shows the evolution of the spreading of the Ga drop as the temperature increased for the different Ga holders analysed. Through the optical window, located at one end of the CVD reactor, we were able to record the evolution of the Ga drop during the deposition process.

Figure 2(a) shows the spreading of the Ga drop on the flat plate holder. Up to 1177 K the GaN drop remained unaffected. From that moment, some cracks appeared at the bottom part of the Ga drop, marked with green arrows. The cracks appear in what it seems to be a solid layer formed covering the Ga drop, as a result of the continuous NH_3_ flow through the reactor at high temperatures. From these cracks, the liquid Ga started to spread out when the furnace reached a temperature of 1203 K. Finally, at around 30 min after the furnace reached 1203 K we observed a scalation of the material covering the crucible, marked with orange arrows in the figure, identified later as GaN by XRD.

Figure 2(b) shows the evolution of the Ga drop on the half of a cylindrical tube holder. The process is similar to that observed in the flat plate holder, although, the spreading process happened at lower temperatures. The cracks at the bottom of the Ga drop started to appear at 1170 K, and at 1203 K, the meniscus of the liquid Ga passed from a convex shape to a concave shape. From that moment, the liquid Ga climbed through the walls of the holder and spread on the quartz liner tube. This caused the leaning of the Ga holder at around 30 min after reaching this temperature. Finally, the scalation of the material covering the crucible occurred at around 32 min after the furnace reached 1203 K, later than in the previous case. All these observations seems to indicate that the deposition process lasts longer when using this Ga holder than when a flat plate holder was used.

When using the concave holder (see Fig. 2(c)), despite the Ga drop followed a similar behaviour as in the previous cases, it was more challenging to see clearly what was happening inside the Ga holder. We could roughly see that at 1201 K two cracks appeared at the bottom of the drop latter than in the two previous cases, and just before the wetting of the crucible by Ga. What we could clearly see was that when the Ga drop reached a temperature of 1203 K it started to climb through the walls of the holder and it spread over the quartz liner. The lean of the Ga holder was observed 44 min after the beginning of the experiment, and the scalation of material was observed at 57 min, much latter than when the half of a cylindrical tube was used.

**Figure 3:**
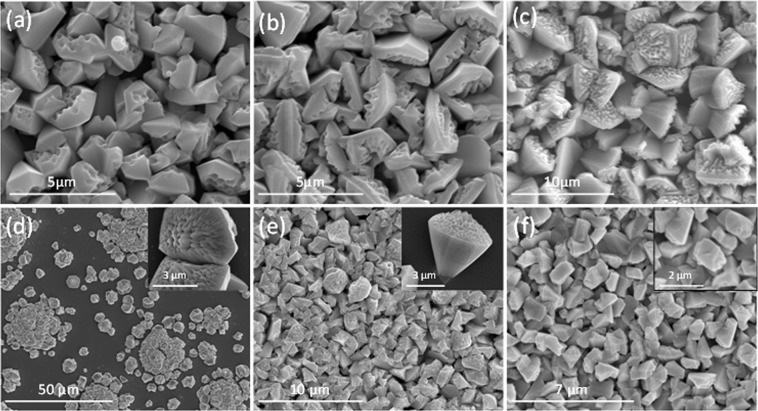
SEM pictures of the nanoporous GaN coating layers obtained on Si (100) substrates using different Ga holders: (**a**) flat plate, (**b**) half cylindrical tube, and (**c**) concave crucible, and different amounts of Ga: (**d**) 0.2 g, (**e**) 0.4 g, and (**f**) 0.6 g.

SEM pictures of the nanoporous GaN coating layers deposited on Si (100) substrates covered with an ethanolic solution of Ni(NO_3_)_2_ using different Ga holders are shown in Fig. 3(a–c). In all cases, the temperature of the reaction was chosen to ensure the total spread of the Ga drop, generating the maximum extension of the surface of metallic Ga to favour its evaporation. The nanoporous GaN coating layers obtained when using a flat plate or a half cylindrical tube as Ga holders were similar, with particles with sizes between 2–3 µm, and a similar degree of porosity (see Fig. 3(a,b)). Nevertheless, the coatings obtained with the half-cylindrical tube have a more uniform thickness. From another side, the particles obtained using the concave crucible (see Fig. 3(c)) show bigger sizes (around 4 µm), and the pores seem to be smaller. The better uniformity obtained for the layers deposited when using a half cylindrical tube might correspond to the fact that the cracks from which the spread of the Ga drop started, occured at earlier temperatures, together with the observation of the scalation process at latter times. This implies that the deposition process lasted longer, contributing to form a more uniform film.

Since by using a half cylindrical tube we obtained more uniform layers, what is a necessary condition for the deposition of antireflective coatings on Si substrates, in the rest of the experiments, we used this kind of Ga holder.

### Effect of the initial Ga amount

Three sets of experiments using different initial amounts of Ga were carried out to analyze its effect on the coating of the Si (100) substrates, while keeping constant the other reaction parameters. Obviously, by changing the initial amount of Ga, what we are doing is changing its concentration in relation with NH_3_, that is maintained constant in all the experiments. Figure 3(d–f) shows SEM pictures of the nanoporous GaN coating layers obtained. In the three cases, particles with different sizes were observed. However, and more importantly, the amount of Ga used affected specially to the degree of coating of the substrate.

When 0.2 g of Ga were used (see Fig. 3(d)) a low degree of coating of the substrate was achieved. This is attributed to the low quantity of Ga evaporated and deposited on the substrate, forming a small number of nucleuses on the substrate. This was confirmed by the fact that the GaN particles were bigger in diameter than in the other cases, around 5–6 µm. A larger surface of the substrate was coated when 0.4 g of Ga were used (see Fig. 3(e)). In contrast with the previous case, the particle diameters are smaller, of around 2 µm. This is attributed to the higher quantity of Ga evaporated and the formation of a higher number of Ga-metallic catalyst nucleuses, which leaded to a larger surface of the substrate coated with smaller nanoporous GaN particles. Finally, in Fig. 3(f) we can see a SEM image of the nanoporous GaN coating layer obtained using 0.6 g of Ga. A similar density of particles and sizesis observed when compared to the experiment using 0.4 g of Ga. However, the shape of the particles is not as obvious as in the previous cases, their sizes are smaller, and the porosity is not as evident. Thus, from the image, it seems that even a higher number of nucleuses were formed in this case, contributing to the reduction in size of the particles. This reduction in size seems to affect also the porosity. These results seem to indicate that it exists an optimum amount of Ga that provides an equilibrium between the number of particles formed, to ensure a good coating of the substrate, and their sizes, to favour the formation of porosity in them. Thus, for the following experiments, the amount of Ga was kept constant at 0.4 g.

### Effect of the deposition time

We performed additional experiments to analyse the influence of the deposition time in the shape and porosity of the GaN particles that form the coating layers. The deposition times selected were 30, 45, 60 and 120 min. We selected these deposition times according to the different events observed in the images shown in Fig. 2. The first one (30 min) is related to the leaning of the crucible when a half cylindrical tube was used, due to the wetting of both, the crucible and the liner. The second one is the time corresponding to the scalation of the material covering the crucible, identified later as GaN by X-ray diffraction, 45 min after the reactor reached 1203 K. Two longer times were explored (60 and 120 min), to analyze if the chemical reaction continued after we observed the scalation of GaN on the crucible.

**Figure 4:**
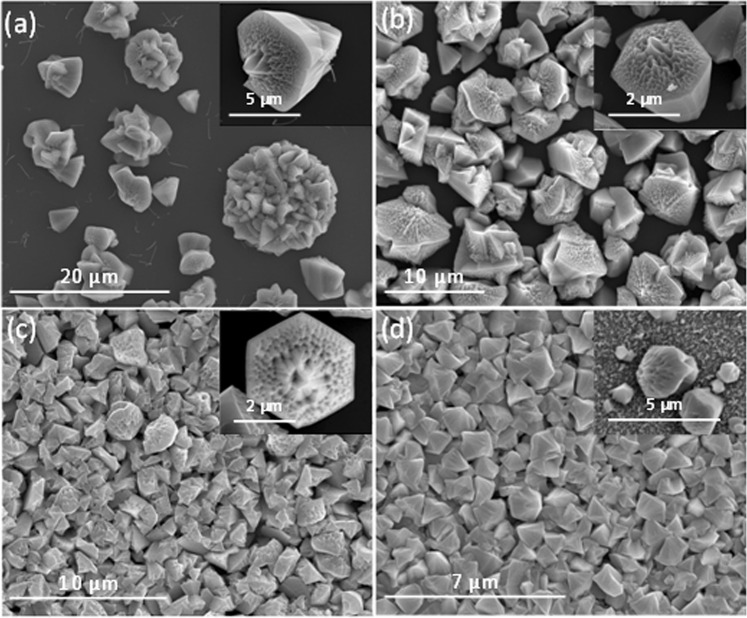
SEM pictures of the particles obtained at (**a**) 30, (**b**) 45, (**c**) 60, and (**d**) 120 min deposition times. Insets show details of individual particles observed.

The deposition time has an important effect on the coating of the substrate with nanoporous GaN particles, as can be seen in Fig. 4, while the size of the particles that form the coating layers is similar. In these images, we can see that the density of nanoporous GaN particles on the surface of the substrate increased when the deposition time increased, until a deposition time of 60 min. From there, it seems that no additional particles nucleated and the particles density remained constant. However, as we increased the deposition time, the morphology of the particles seems to fade away (see Fig. 4(d)). Smaller particles can be seen covering the substrate. This might be due to the decomposition of the previously formed particles, due to the high temperature and low pressure at which the sample is exposed during the growth experiment, and the further nucleation of smaller particles at expenses of the bigger ones. The particles obtained, however, follow the same porous pattern observed up to now: nanopores located only on the (0002) face, with wider pores in the central part of the particles^22^.

Thus, for the rest of the experiments we decided to use a deposition time of 60 min.

### Effect of the catalyst

To deposit nanoporous GaN films on Si substrates, it is necessary to coat the substrate with a metallic catalyst^21^. Here, we tested how, different catalysts, affected to the formation of these films: Ni, introduced as Ni(NO_3_)_2_ dissolved in ethanol, and 20 nm thick films of Ni, Au, Pt, and Ti, all of them deposited by RF sputtering.

**Figure 5:**
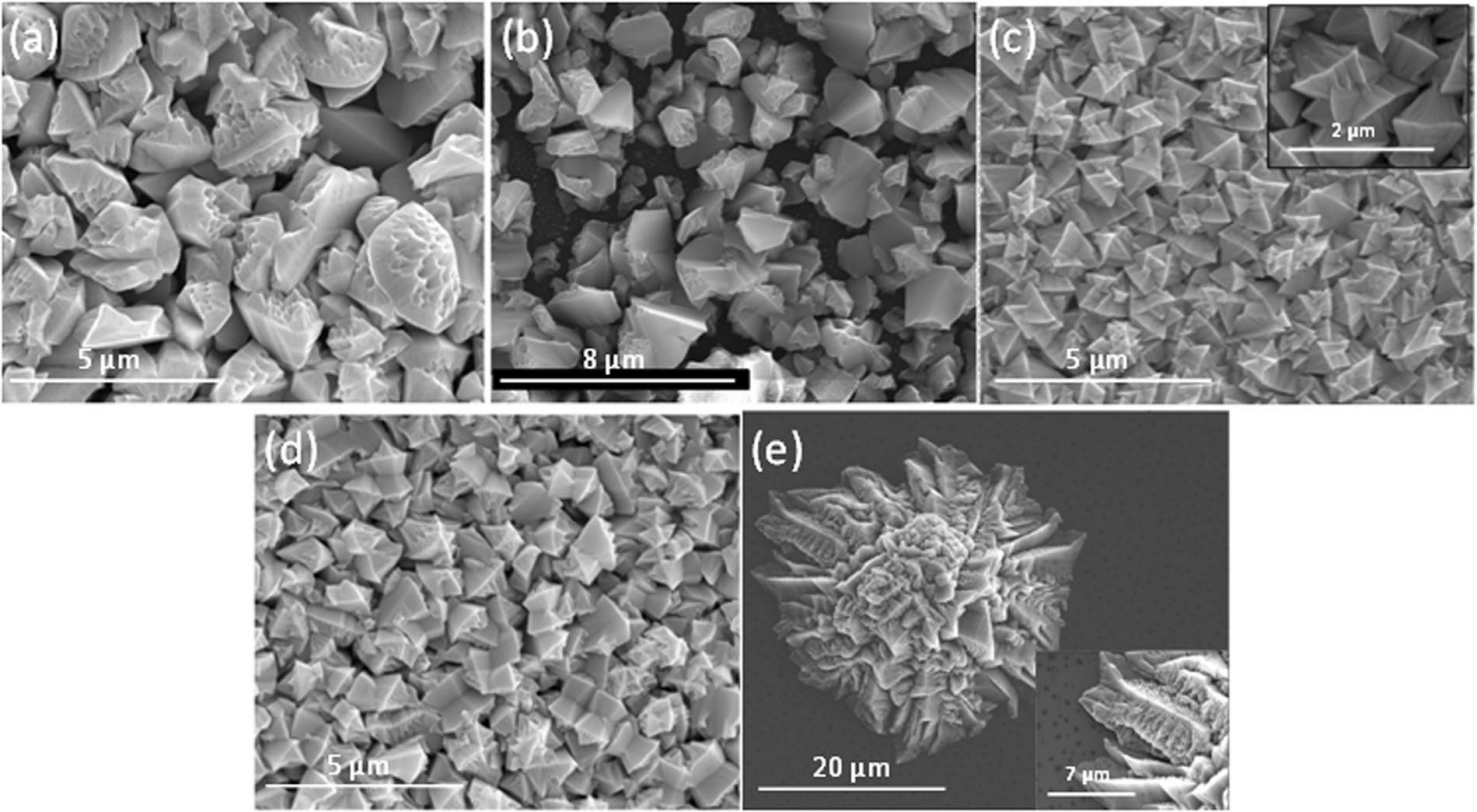
SEM pictures of nanoporous GaN coating layers deposited on Si (100) coated with different catalysts: (**a**) Ni(NO_3_)_2_, (**b**) Ni, (**c**) Au, (**d**) Pt, and (**e**)Ti.

SEM pictures of the deposited nanoporous GaN films on Si (100) substrates, shown in Fig. 5, demonstrate that in all cases, GaN appears in the form of micron-sized nanoporous particles. The bigger particles with the highest degree of porosity were obtained using Ni(NO_3_)_2_ as catalyst. It is curious to notice that Ni, introduced as Ni(NO_3_)_2_ or directly as metallic Ni has a different effect on the morphology and porosity degree of the particles, probably related to the distribution of the catalyst on the surface of the substrate, despite Ni(NO_3_)_2_ is reduced to Ni, under the reaction conditions^21^. While bigger particles with a higher level of porosity were obtained using Ni(NO_3_)_2_, the particles grown on a continuous Ni film had an irregular shape and a lower degree of porosity (see Fig. 5(a,b)). Smaller porous GaN particles were obtained when using Pt and Au catalysts, very homogeneous in size and exhibiting a uniform coating on the substrate (see Fig. 5(c,d)).

**Figure 6:**
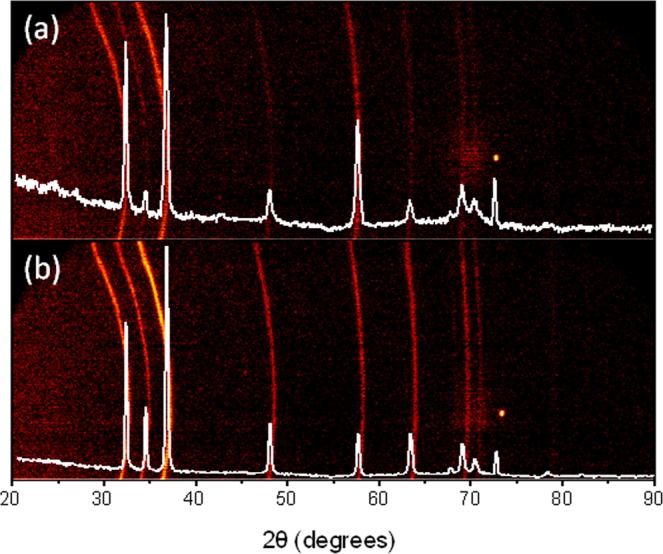
X-ray diffraction patterns and Debye rings of the nanoporous GaN films deposited on Si (100) substrates coated with (**a**) Ti and (**b**) Ni(NO_3_)_2_.

Nanoporou sGaN coating layers deposited using Ti as catalyst have a totally different morphology, remembering the shape of a sea star. Apparently, GaN started to grow as an epitaxial layer on the substrate with a further growth of nanoporousGaNparticles on the top of it (see Fig. 5(e)). This texturation of the film, induced by the catalyst, is confirmed by the XRD pattern recorded for this sample shown in Fig. 6. The non-uniform distribution of the intensity in the Debye rings recorded with the GADDS detector (see Fig. 6(a)) and the different intensity of the diffraction peaks, indicated this texturation. However, it is not easy to establish which is the preferential orientation of the particles from the data obtained. What we observed is that the (0002), $(10\bar{1}3)$ and $(20\bar{2}1)$ reflections showed a lower intensity than expected. This texturation induced by the catalyst is more evident when we compare this XRD pattern with that of the nanoporous GaN particles obtained using Ni(NO_3_)_2_ as catalyst (see Fig. 6(b)), that follows the same trend indicated in the reference pattern.

**Figure 7:**
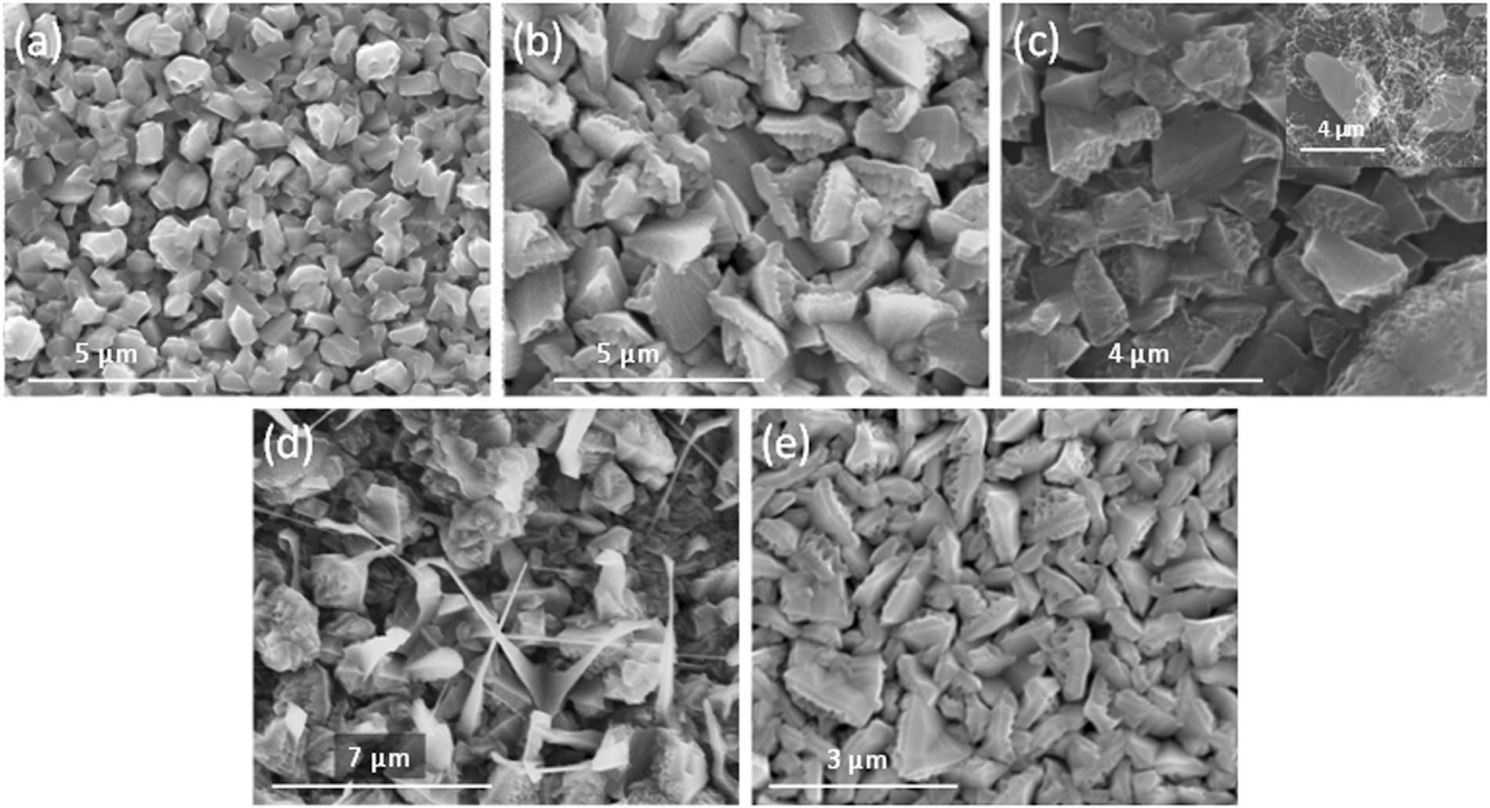
SEM pictures of nanoporous GaN films deposited on Si (111) substrates coated with different catalysts: (**a**) Ni(NO_3_)_2_, (**b**) Ni, (**c**) Au, (**d**) Pt, and (e) Ti.

The results obtained when using Si (111) substrates, instead of Si (100) substrates, are very similar, with particles with a mean size of 2–5 μm (see Fig. 7). However, the GaN particles obtained on Si (111) substrates tended to be more irregular in shape. In addition, in the case of using Ti as catalyst, we did not observe the texturation effect obtained when using Si (100) substrates, as can be seen in Fig. 7(e).

**Figure 8:**
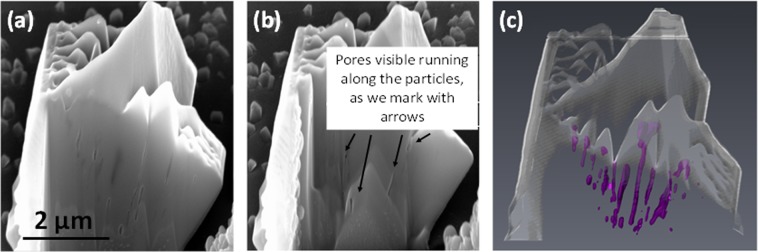
(**a**,**b**) SEM images of a nanoporousGaN particle grown on a Si (100) substrate using Ni(NO_3_)_2_ as catalyst, obtained at two different times of slicing with FIB. (**c**) Reconstruction of the internal nanoporous structure of the same particle after processing all the images recorded during the slicing process with FIB, including the original external shape of the particle to illustrate the distribution and organization of the nanopores.

Thus, the coatings obtained can be described as formed by GaN particles with smooth lateral faces and internal pores contained only on their basal plane, as confirmed by the SEM images obtained by focused-ion-beam (FIB) slicing of one of these GaN particles, shown in Fig. 8(a,b), recorded at two different moments of the slicing process. By processing all the images recorded during the FIB slicing process, we could generate an image illustrating the internal distribution of these pores (see Fig. 8(c)), demonstrating that they are straight, that they do not cross each other, and that their internal diameters are more or less constant, only broadening when they arrive to the surface of the particles.

**Figure 9:**
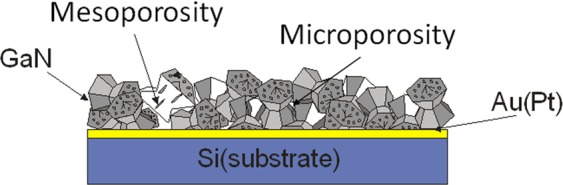
Scheme showing the dual porosity (microposity and mesoporosity) of the nanoporousGaN layers produced on Si (100) and Si (111) substrates.

With all this information, the GaN layers generated can be seen both as microporous and mesoporous, since the microporosity would stem for the free space between particles and the mesoporosity would arise from the internal porosity^23^ as described in the scheme shown in Fig. 9.

After the analysis of the different deposition parameters, the optimum parameters for the coating of Si substrates with these nanoporousGaNpolycrystalline layers seem to be: (i) using a Ga holder with the shape of a half cylindrical tube; (ii) using an initial amount of Ga of 0.4 g; (iii) undertaking the deposition reaction during 60 min, and (iv) coating the Si substrates with Au or Pt layers acting as catalysts.

### Reflectance measurements

**Figure 10:**
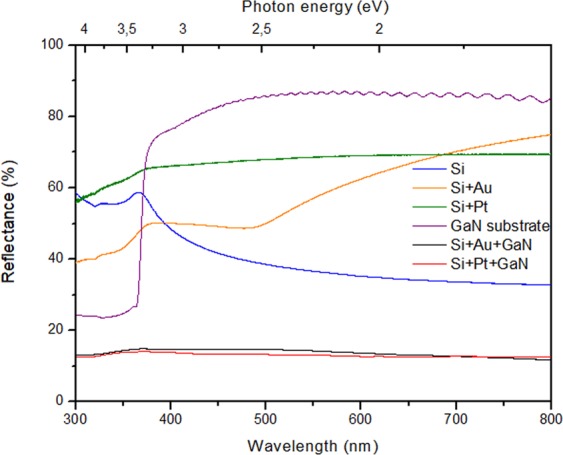
Reflectance spectra of (blue) Si substrate, Si substrate coated with a 20 nm thick layer of (green) Pt and (orange) Au nanoporousGaNparticles grown on Si substrates coated with a layer 20 nm thick of (black) Au and (red) Pt, and (purple) flat GaN substrate.

To probe the potentially of these nanoporousGaNcoating layers on Si substrates, we collected their reflectance spectra. Figure 10 shows the reflectance spectra of the nanoporousGaNparticles layers grown on Si (100) substrates coated with a layer of Auand Pt, 20 nm thick. The reflectivity of bare Si is about 40% in the wavelength range of 400–800 nm, which is consistent with previous reports^24^, whereas the Au covered Si substrates have a reflectance value between 50–70% in the wavelength range of 500–800 nm, and those coated with Pt have a reflectance of around 70% in the range of 400–800 nm, being a little smaller at shorter wavelengths. In fact, the increase in the reflectance values of the Si substrates coated with Au and Pt are due to the homogeneous and continuous metallic layer film with which they are coated^25^. Coating these substrates with a layer of nanoporous GaN particles decreased substantially their reflectivity to values around 10%. The decrease in reflectivity can be attributed to the high surface area of the nanoporous particles combined to the fact that the diameters of the pores lie in the nanoscale subwavelength range, promoting a similar effect to that previously observed in GaN nanorod arrays grown of Si substrates^26^.

If we compare our results with those previously obtained for non-porous GaN particles deposited on Si (111) substrates^24^, they are similar, but with the advantage of having a flat response in the whole spectral range investigated in our case. When compared to the use of GaN nanorod arrays grown on Si substrates^26^, our reflectance response is better for wavelengths above 400 nm. When compared with other technologies used to fabricate antireflective coatings on Si substrates, we obtained similar values of reflectance than those obtained using porous black silicon^27^ at wavelengths below 400 nm and above 600 nm, but again with the advantage of having a constant value of reflectance over the whole spectral range analysed in our case. The improvement of the antireflective properties of the coatings we developed is also evident when comparing their performance and that of textured Si substrates with pyramidal hillocks^28^, especially in the range of wavelengths below 500 nm. Finally, by comparing our results with those based on interference-type antireflective coatings^29^, we obtained an improved reflectance for wavelengths shorter than 400 nm. Thus, in general we can conclude that our antireflective coatings based on nanoporous GaN particles show an improvement on current antireflective coatings deposited on Si substrates, especially at wavelengths below 400–500 nm.

It has been pointed out the interest of such improved antireflective properties in the UV for enhanced efficiency in Si-based photovoltaic cells when using, for instance, SnO nanobelt structures electrodeposited on Si substrates^30^. However, these results are especially important for the development of UV photodetectors. Up to now, antireflective properties in GaN-based UV photodetectors have been given by the deposition of ZnOnanorod arrays on the surface of GaN^31^, or by attaching a polymer layer with an array of micropyramidal structures on GaN surfaces^10^. In the first case, a reduction of 94% of the reflectance in average was observed in the UV (200–400 nm) and 77% in the visible (400 to 800 nm), which resulted in an enhanced sensitivity of the photodetectors operating at 365 nm from 136.3% to 148.2%, depending on the temperature^31^. In the second case, although the reduction in reflectance was smaller (20% in the UV and 43% in the visible), it still allowed to increase the sensitivity of the UV sensor by 48.5%^10^. In our case, we observed a reduction of the reflectance of 40% in the UV and 83% in the visible when compared to flat GaN. So, these results seem to indicate that the polycrystalline GaN films with dual porosity we produced might be a good solution to enhance the sensitivity of UV photodetectors without incorporating an extra nanostructured material, simplifying the fabrication process of these devices. Furthermore, the metallic layer used as catalyst to facilitate their deposition, can be used as the metallic electrode located under the sensor, as we proofed previously for the electric characterization of such films^32^, allowing for an easier and cheaper fabrication of this kind of devices.

### Surface wettability characterization

**Figure 11:**
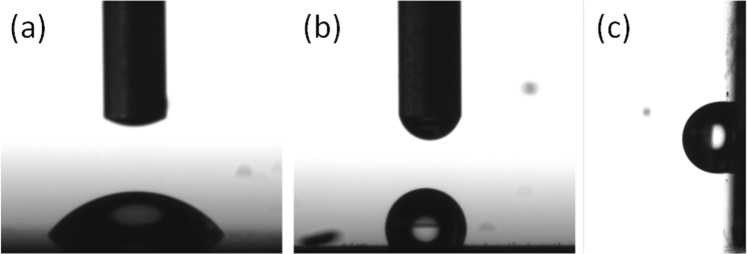
Picture a 2 μl droplet of water deposited on (**a**) a bare Si (100) substrate, (**b**) the same substrate coated with a layer of polycrystalline nanoporous GaN used to determine the static contact angle. (**c**) Picture used used to determine the advancing and recessing contact angles in the sample shown in (**b**) tilted by 90°.

Since the presence of hierarchical roughness on the coatings, such as that present in our microporous and mesoporous GaN films allows generating hydrophobic surface because of the trapped air pockets between the water droplets and the surface of the films following the Cassie-Baxter model^6^, we investigated the surface wettability properties of our nanoporous GaN antireflective coatings. The root mean square roughness (RMS) of our films, measured by a Sensofar PLµ-2300 confocal microscope in an area of 1.55 mm^2^, is ∼200 nm. Figure 11 shows the static contact angle (CA) for the surface of a bare Si (100) substrate (Fig. 11(a)), and the surface of a Si (100) substrate coated with our nanoporous GaN layer (Fig. 11(b)). As can be seen in the image, the contact angle of the water droplet increases substantially from 57° ± 0.7° for the bare Si substrate to 119° ± 3° when the Si substrate is coated with a layer of nanoporous GaN particles. This indicates the hydrophobic character of the coatings we developed, although they did not reach the superhydrophobic regime. We can appreciate a slightly asymmetry between the apparent contact angle (ACA) determined from the left and right sides of the water droplet due to the inhomogeneous roughness along the surface^33^ leading to a deviation of around 3° in the measured ACA. The contact angle of the deposited nanoporous GaN layer is comparable to those obtained in self-organized GaN nanoflowers (111°)^15^ but still far from those obtained on GaN microbelts (150°)^17^ and nanowires (155°)^34^ due to the lower ability of our sample to trap air between the water droplet and the nanoporous GaN layer, when compared to these systems. Also, we reached a higher contact angle value than the one reported in nanostructured N-polar GaN chemically functionalized with lauric acid (ref). Thus, we believe that if we functionalize chemically the surface of our microporous and mesoporous GaN films, we might reach the superhydrophobic regime.

Figure 11(c) shows the deposited water droplet on a nanoporous GaN films deposited on a Si (100) substrate tilted by 90° to obtain the contact angle hysteresis (CAH) by measuring the advancing and the recessing contact angle. The obtained contact angle hysteresis is only 4.5°, confirming that the wetting mechanism is described by the Cassie-Baxter model^35^. This indicates that these porous films can be used in self-cleaning applications.

Since we have a rough surface, and according to the literature, we cannot apply the Young’s equation to estimate the intrinsic contact angle and extract from it the surface energy of our porous film^33^. This is because the wetting mechanism is not only afected by the surface energy but also for the roughness^34^. Previously in the literature, the surface tension for flat GaN has been calculated to be γ_SV_ = 1.89 N·m^−1^ ^35^. Such a high surface tension would correspond to a wetting surface, i.e. a hydrophilic behaviour^17^. Instead, our surface shows a hydrophobic character due to the air trapped within the surface protrusions^17^, consistent with the Cassie-Baxter model we assumed.

## Conclusions

We optimized the deposition of nanoporous GaN films on Si substrates by analyzing how different reaction parameters, such as the shape of the Ga holder, the initial Ga amount, the deposition time, and the catalyst affected on the morphology, size and porosity degree of these layers grown by the direct reaction of metallic Ga and NH_3_ in a CVD system. The optimum conditions for the deposition of such coatings are the use of a Ga holder with the shape of a half cylindrical tube with the following conditions: an initial Ga amount of 0.4 g; a reaction time of 60 min, and using Au or Pt as catalysts, while keeping the temperature of the reaction fixed at 1200 K, the flow of ammonia at 75 sccm and the pressure at 15 Torr.

Under these conditions, we have been able to coat Si substrates with a layer of nanoporous GaN particles that proved to be antireflective, reducing the reflectance of Si or flat GaN by 50% or more, improving the results obtained by previous developed technologies, especially in the short wavelengths range, below 400–500 nm. This is especially interesting to use these porous GaN films as UV sensors.

Furthermore, these nanoporous layers probed to be hydrophobic as fabricated, with any post-growth treatment or chemical functionalization, contributing to the durability of such antireflective coatings due to its induced self-cleaning effect.

Taking into account that photocatalysis activity has already been reported for GaN to photodegrade organic dyes^36^, or for solar-to-hydrogen conversion^37^, and the combination of its chemical stability at elevated temperatures and its hardness, we believe that in the future new applications will be developed for these mechanically and chemically robust polycrystalline nanoporous GaN multifunctional coatings. But a field in which we believe they might play an important role is in the development of UV sensors with enhanced sensitivity, purely fabricated with GaN, avoiding the incorporation of alien materials as it has been done up to now, simplifying at the same time the fabrication process of these devices.
